# The Use of Indocyanine Green Fluorescence Angiography in Pediatric Surgery: A Systematic Review and Narrative Analysis

**DOI:** 10.3389/fped.2021.736242

**Published:** 2021-09-13

**Authors:** Annie Le-Nguyen, Maeve O'Neill Trudeau, Philippe Dodin, Mark R. Keezer, Christophe Faure, Nelson Piché

**Affiliations:** ^1^Department of General Surgery, Université de Montréal, Montréal, QC, Canada; ^2^Department of Pediatric Surgery, Centre Hospitalier Universitaire Sainte-Justine, Université de Montréal, Montréal, QC, Canada; ^3^Centre Hospitalier Universitaire Sainte-Justine, Université de Montréal, Montréal, QC, Canada; ^4^Department of Neurosciences, Université de Montréal, Montréal, QC, Canada; ^5^Centre Hospitalier de l'Université de Montréal (CHUM), Montréal, QC, Canada; ^6^School of Public Health, Université de Montréal, Montréal, QC, Canada; ^7^Department of Gastroenterology, Hepatology, and Nutrition, Centre Hospitalier Universitaire Sainte-Justine, Université de Montréal, Montréal, QC, Canada

**Keywords:** colorectal anastomosis, pediatric surgery, indocyanine green, fluorescence angiography, intraoperative assessment, perfusion

## Abstract

**Purpose:** Indocyanine green fluorescence angiography (ICG-FA) is a validated non-invasive imaging technique used to assess tissue vascularization and guide intraoperative decisions in many surgical fields including plastic surgery, neurosurgery, and general surgery. While this technology is well-established in adult surgery, it remains sparsely used in pediatric surgery. Our aim was to systematically review and provide an overview of all available evidence on the perioperative use of indocyanine green fluorescence angiography in pediatric surgical patients.

**Methods:** We conducted a systematic review with narrative synthesis in conformity with the PRISMA guidelines using PubMed, Medline, All EBM Reviews, EMBASE, PsycINFO, and CINAHL COMPLETE databases to identify articles describing the perioperative use of ICG-FA in pediatric patients. Two independent authors screened all included articles for eligibility and inclusion criteria. We extracted data on study design, demographics, surgical indications, indocyanine green dose, and perioperative outcomes. We developed a risk of bias assessment tool to evaluate the methodological quality of included studies.

**Results:** Of 1,031 articles retrieved, a total of 64 articles published between 2003 and 2020 were included reporting on 664 pediatric patients. Most articles were case reports and case series (*n* = 36; 56%). No adverse events related to ICG-FA were reported in the included articles. Risk of bias was high. We did not conduct a meta-analysis given the heterogeneous nature of the populations, interventions, and outcome measures. A narrative synthesis is presented.

**Conclusion:** Indocyanine green fluorescence angiography is a safe imaging technology and its use is increasing rapidly in pediatric surgical specialties. However, the quality of evidence supporting this trend currently appears low. Case-control and randomized trials are needed to determine the adequate pediatric dose and to confirm the potential benefits of ICG-FA in pediatric surgical patients.

**Systematic Review Registration:** This study was registered on Prospero a priori, identifier: CRD42020151981.

## Introduction

Indocyanine green fluorescence angiography (ICG-FA) is an imaging technology that allows real-time visualization of tissue perfusion (1). With a half-life of 3 to 5 min, ICG allows repeated injections during a procedure (1). Adverse events have been reported in 1 out of 42,000 patients and include anaphylactic reactions in patients with iodine allergy, making this a contraindication for ICG use (2). Initially, this dye was intended for use in ophthalmic angiography, cardiac output measurements, and hepatic function studies (3). In the early 2000s, the interest in ICG-FA importantly increased. Current surgical applications in adults include tumor detection, assessment of flap perfusion, and fluorescence imaging of tissue and organ perfusion in colorectal and hepatobiliary surgeries (1). Recently, the use of indocyanine green fluorescence angiography in the pediatric population has rapidly gained in popularity. We performed a systematic review and narrative synthesis of the literature to capture and analyze all available evidence on the use of ICG-FA in pediatric surgical patients. The objective of this review was to determine if current data support its use.

## Materials and Methods

We conducted a comprehensive systematic review in conformity with the PRISMA (Preferred Reporting Items for Systematic Reviews and Meta-Analyses) guidelines (4). Institutional review board approval was not necessary. Before data extraction, we registered our systematic review protocol with PROSPERO (ID number CRD42020151981). Six electronic databases were systematically searched in December 2019 with an updated search on January 14, 2021: PubMed (NLM), Medline (Ovid), All EBM Reviews (Ovid), EMBASE (Ovid), PsycINFO (Ovid), and CINAHL COMPLETE (EBSCO). The search strategy was designed in collaboration with a senior hospital librarian (PD) to identify all relevant articles reporting the use of ICG-FA in the pediatric population. There was no restriction to time, language, study subjects, and type of articles. References of included articles were hand-searched to identify additional relevant studies.

### Study Selection and Data Extraction

Two authors (ALN and MOT) independently screened all titles and abstracts. When potentially relevant, the two authors independently screened all full texts to decide which articles to include in the final review. Any disagreements were resolved by consensus or with the help of the senior author (NP). Studies were included in the analysis if they: (1) included patients <18 years of age and (2) focused on the perioperative use of ICG-FA. We excluded reviews, letters to the editors, editorials, commentaries, abstracts, animal and adult studies, as well as ophthalmological surgical articles. Articles that included both pediatric and adult patients were included when the mean or median age was < 18 years old, or when enough data on pediatric patients allowed a separate analysis.

We classified studies as descriptive studies and analytic studies. Descriptive studies included case series and case reports whereas analytic studies were defined as cohort studies with a comparison group and experimental studies (e.g., randomized controlled trials) (5).

### Study Quality Assessment

We developed a risk of bias tool based on the Newcastle-Ottawa (6), Methodological quality and synthesis of case series and case reports (7), and MINORS (Methodological Index for Non-Randomized Studies) tools (8). Additional support from an epidemiology expert (MRK) was obtained for selecting criteria and developing the risk of bias tool (see Table 1). Two reviewers (ALN and MOT) independently evaluated the risk of bias of every included study and reported them as high, low, or unclear (Table 2). Disagreements were solved by consensus.

**Table 1:** Risk of bias assessment tool.

**Criteria of risk of bias assessment tool**		
Selection	Q1	Is there a clearly stated research objective or question?
	Q2	Are the demographic and clinical factors of patients clearly described?
	Q3	Is the sample representative of the population of interest?
Data collection	Q4	Were data collected prospectively?
	Q5	Were outcomes appropriate for the study aim?
	Q6	Were outcomes objectively assessed?
	Q7	Was the follow-up period appropriate to the study aim?
Case ascertainment	Q8	Was the dosage and type of indocyanine green specified?
	Q9	Were there sufficient information to allow detailed appraisal of the evidence?
Statistical analysis	Q10	Were adequate statistical analyses to the study design performed? (Are potential confounders adequately controlled for?)
If comparative study	Q11	Was there an adequate clearly defined control group?
	Q12	Were both comparative cohorts equivalent?

**Table 2:** Risk of bias of included studies.

**References**	**Selection**			**Data collection**				**Case ascertainment**		**Stats**	**If comparative study**	
	**Q1**	**Q2**	**Q3**	**Q4**	**Q5**	**Q6**	**Q7**	**Q8**	**Q9**	**Q10**	**Q11**	**Q12**
Ambekar et al. (9)											N/A	N/A
Asayama et al. (10)	N/A										N/A	N/A
Aung et al. (11)	N/A										N/A	N/A
Bada-Bosch et al. (12)	N/A										N/A	N/A
Bryant et al. (13)	N/A										N/A	N/A
Calabro et al. (14)											N/A	N/A
Chang et al. (15)	N/A										N/A	N/A
Chen-Yoshikawa et al. (16)	N/A										N/A	N/A
Cheng et al. (17)											N/A	N/A
Chung et al. (18)	N/A										N/A	N/A
Cleveland et al. (19)	N/A										N/A	N/A
Connolly et al. (20)	N/A										N/A	N/A
Drobot et al. (21)	N/A										N/A	N/A
Esposito et al. (22)											N/A	N/A
Esposito et al. (23)												
Esposito et al. (24)											N/A	N/A
Esposito et al. (25)												
Esposito et al. (26)											N/A	N/A
Esposito et al. (27)											N/A	N/A
Fernandez-Bautista et al. (28)	N/A										N/A	N/A
Fung et al. (29)	N/A										N/A	N/A
Greives et al. (30)	N/A										N/A	N/A
Guillen et al. (31)												
Herz et al. (32)											N/A	N/A
Hinchcliff et al. (33)	N/A										N/A	N/A
Hirayama et al. (34)											N/A	N/A
Horie et al. (35)											N/A	N/A
Hori et al. (36)											N/A	N/A
Iinuma et al. (37)	N/A										N/A	N/A
Ishikawa et al. (38)											N/A	N/A
Kaneshi et al. (39)	N/A										N/A	N/A
Kato et al. (40)	N/A										N/A	N/A
Kato et al. (41)	N/A										N/A	N/A
Kato et al. (42)											N/A	N/A
Kim et al. (43)	N/A										N/A	N/A
Kitagawa et al. (44)											N/A	N/A
Kogon et al. (45)											N/A	N/A
Martins et al. (46)												
Mihara et al. (47)											N/A	N/A
Mitani et al. (48)	N/A										N/A	N/A
Nossek et al. (49)	N/A										N/A	N/A
Ogata et al. (50)	N/A										N/A	N/A
Otake et al. (51)	N/A										N/A	N/A
Pourmoghadam et al. (52)	N/A										N/A	N/A
Rentea et al. (53)											N/A	N/A
Sanchez-Fernandez et al. (54)	N/A										N/A	N/A
Shafy et al. (55)											N/A	N/A
Shibasaki et al. (56)											N/A	N/A
Shirota et al. (57)	N/A										N/A	N/A
Shirotsuki et al. (58)											N/A	N/A
Souzaki et al. (59)											N/A	N/A
Sugimoto et al. (60)	N/A										N/A	N/A
Tan et al. (61)	N/A										N/A	N/A
Tanabe et al. (62)											N/A	N/A
Takagi et al. (63)	N/A										N/A	N/A
Takahashi et al. (64)	N/A										N/A	N/A
Tomioka et al. (65)	N/A										N/A	N/A
Tsuzuki et al. (66)	N/A										N/A	N/A
Ueba et al. (67)	N/A										N/A	N/A
Vogt et al. (68)											N/A	N/A
Yada et al. (69)	N/A										N/A	N/A
Yamamichi et al. (70)											N/A	N/A
Yanagi et al. (71)												
Yokoyama et al. (72)	N/A										N/A	N/A

*Green, low bias risk; Red, High bias risk; yellow, unclear bias risk*.

### Statistical Analysis

We did not perform a meta-analysis given the important heterogeneity of included studies. However, narrative synthesis is presented to guide clinicians with the current pediatric surgical indications and doses of indocyanine green angiography.

## Results

**Figure 1:**
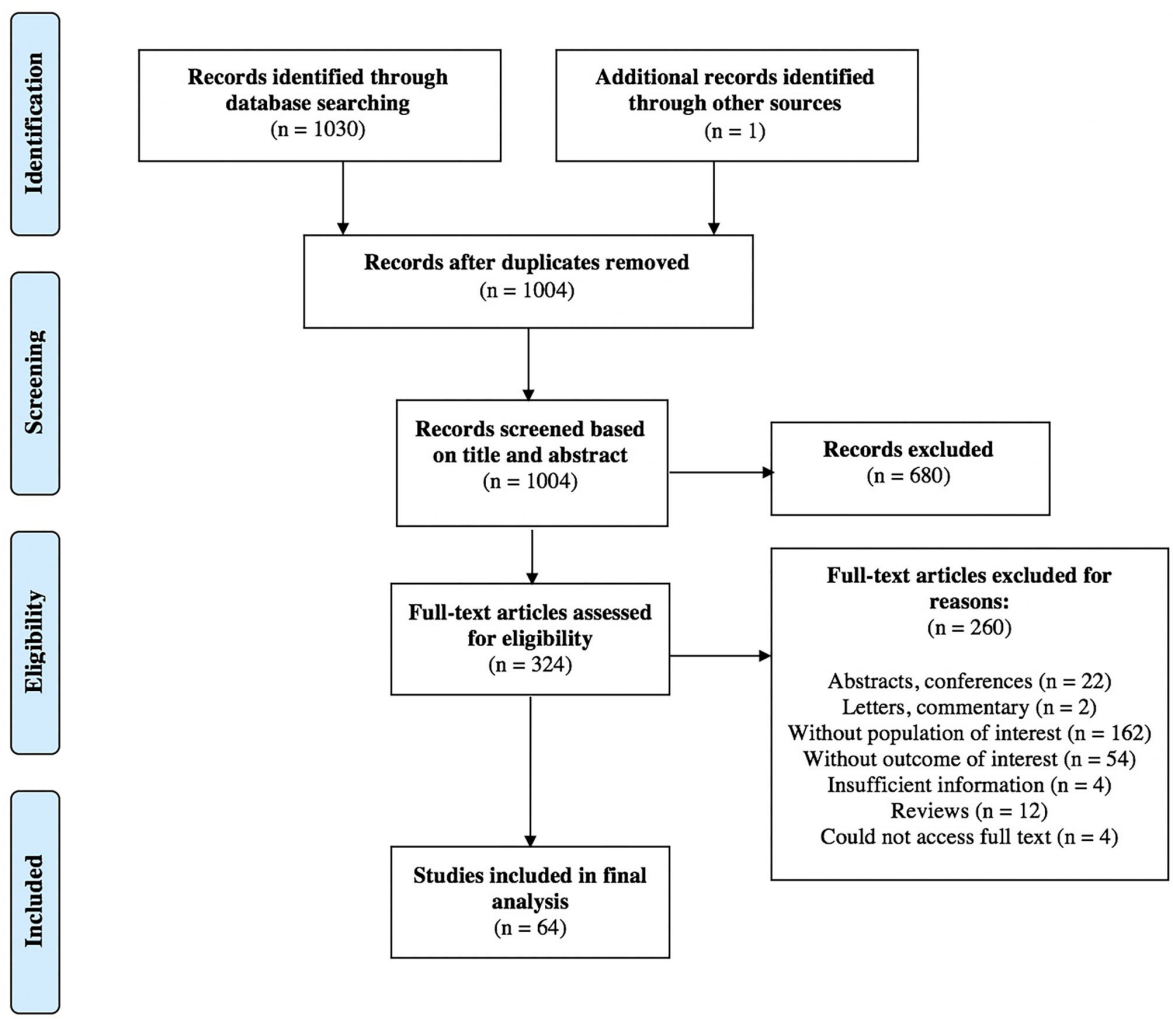
PRISMA flow diagram of selection of studies for inclusion in the systematic review.

The selection process of articles and reasons for study exclusion are reported in a PRISMA flowchart (Figure 1). A total of 937 articles were retrieved through the initial database search, and 93 were additionally identified in January 2021. One study was included after hand-searching references (53). Sixty-four articles, including 664 pediatric patients, from 2003 to 2020 met the inclusion criteria for narrative synthesis in our systematic review. Case reports and case series represented 38 and 19% of included studies, respectively. The remaining 43% were retrospective and prospective studies with no randomized controlled trials. Table 3 presents an outcome summary.

**Table 3:** Study outcome summary separated in different surgical specialties.

**References**	**Study design** **(data collection)**	**Indication** **(Country)**	**Number children/adults**	**Age**	**ICG dose,** **route administration, timing**	**Outcomes**
**Pediatric surgery (** ***n*** **= 29)**						
Bada-Bosch et al. (12)	Descriptive study	Splenic cyst (Spain)	1	13 y	0.2 mg/kg IV, intraoperatively	Guides laparoscopic partial splenectomy
Bryant et al. (13)	Descriptive study	Gallbladder duplication (United States)	1	17y	Not specified	Not able to provide adequate details to assist in dissection
Calabro et al. (14)	Descriptive study (prospective)	Lap cholecystectomy (United States)	31	6–18y	2.5 mg IV, intraoperatively before surgical incision	Useful for Calot triangle dissection
Chang et al. (15)	Descriptive study	Post-operative chylothorax (Taiwan)	1	3m	2 ml of Diagnogreen 0.5% SC, bilateral inguinal	Successful visualization of leak
Chen-Yoshikawa et al. (16)	Descriptive study	Hepatoblastoma lung metastasis (Japan)	1	3y	0.5 mg/kg ICG IV, 24h pre-op	2y without recurrence
Chung et al. (18)	Descriptive study	Hepatocellular carcinoma (China)	1	9y	0.5 mg/kg ICG IV, 24h pre-op	Useful for laparoscopic hepatectomy—identification of tumor, adequate resection margins
Esposito et al. (22)	Descriptive study (ambispective)	Pediatric minimally invasive procedures (Italy)	46	8–18y	Depends on procedure: 0.3–0.5 mg/kg IV (abdominal procedures), 6 mg intra-testicular (varicocelectomy)	Success for lap procedures: cholecystectomy, varicocelectomy, abdominal mass excision, nephrectomy
Esposito et al. (23)	Descriptive study (retrospective)	Lap cholecystectomy (Italy)	215^*^	5–17y	0.4 mg/kg IV, 18h pre-op	Lower average operative time with ICG and quicker to view critical view of safety
Esposito et al. (26)	Descriptive study (ambispective)	Varicocelectomy, nephrectomy, renal cyst deroofing, cholecystectomy, lymphoma and abdominal tumor (Italy)	76	1–18y	0.25–0.5 mg/ml/kg IV, intra-testicular or intra-lesion; 15–18h pre-op for cholecystectomies	No clear demarcation between cystic malformation and normal parenchyma (thoracoscopic lobectomy)
Esposito et al. (27)	Descriptive study (retrospective)	Simple renal cysts (Italy)	13^†^	8y (5–15 y)	0.35 mg/kg IV, intraoperatively	Guides cyst evacuation and deroofing
Fernandez-Bautista et al. (28)	Case series	Lap procedures: aortocoronary fistula, varicocelectomy, cholecystectomy, nephrectomy (Spain)	5	3–14 y	0.2 mg/kg IV	Safe dissection of vascular anatomy
Fung et al. (29)	Descriptive study	Pulmonary nodule (tuberculosis) (China)	1	4y	0.5 ml intra-lesion, 1h pre-op CT-guided	Confirmation of complete excision and localization of nodule
Guillen et al. (31)	Descriptive study (ambispective)	Surgical anatomy (biliary tract, oncology, pulmonary nodules, esophagus and duodenal atresia) (Spain)	20	10.9y (7d−19y)	0.15 mg/kg to 3 mg/kg IV, 24h pre-op to intra-op depending on indication	No complication, relevant information in 90% of the cases
Hirayama et al. (34)	Descriptive study	Kasai procedure for biliary atresia (Japan)	5	31–75d	0.1 mg/kg IV, 24h preop	Intra-op detection of bile leak. Fluorescence detection in feces postop.^‡^
Iinuma et al. (37)	Descriptive study	Intestinal ischemia (Japan)	1	15y	25 mg IV	Detection of abnormal vascular flow
Kitagawa et al. (44)	Descriptive study (ambispective)	Hepatoblastoma with pulmonary metastases (Japan)	10	1–11y	0.5 mg/kg IV, 24h preop	Detection of nodules not seen on palpation or CT scan, but 29/250 false positive nodules
Mihara et al. (47)	Descriptive study (retrospective)	Chylous pleural effusion, ascite (Japan)	8	25d−7m	0.1 ml of Diagnogreen 0.05%, bilateral feet and hands	Useful for lymphography+ lymphaticovenous anastomosis (LVA). *Limitations*: LVA had no effect in 2 patients.
Mitani et al. (48)	Descriptive study	Hepatoblastoma (Japan)	1	32m	0.5 mg/kg IV, 2 days preop	Successful identification of hepatoblastoma, no recurrence at 13m
Otake et al. (51)	Descriptive study	Chylous ascites (Japan)	1	13y	5 ml, popliteal fossa	No recurrence at 6m
Rentea et al. (53)	Descriptive study (retrospective)	Anorectal malformation, cloaca, Hirschsprung (United States)	13	1.9y (0.5–7.8y)	0.2 mg/kg ICG IV	Change in the operative plan in 4/12 (31%)
Shafy et al. (55)	Descriptive study (retrospective)	Colorectal surgeries, cholecystectomy, renal procedures, and more (United States)	100	Median 12 y	0.5–2.5 mg/ml (depending on weight and procedure)	Proved safety with repeated injections of ICG
Shibasaki et al. (56)	Descriptive study	Congenital pleural effusion and ascites (Japan)	10	1–275d	0.25 mg SC	Can be performed at bedside, consistent with clinical course. Change in skin color (n = 1). *Limitations*: only visualize superficial lymphatic vessels (< 2 cm)
Shirotsuki et al. (58)	Descriptive study (retrospective)	Tracheoesophageal fistula (Japan)	10	1–10d	0.025 mg inter-toe injection, 1h preop	
Souzaki et al. (59)	Descriptive study (retrospective)	Hepatoblastoma with pulmonary metastases (Japan)	5	12–36m	0.5 mg/kg IV, 90.5 +/- 33.7 h before hepatectomy/liver transplant and 21.8 +/- 3.4 h before lung resection	Successful detection of 1.2 mm tumors and ad 6 mm from lung surface. *Limitations*: 1 false positive pulmonary lesion
Takahashi et al. (64)	Descriptive study	Recurrent hepatoblastoma with peritoneal metastases (Japan)	1	14 y	0.5 mg/kg IV, 72h preop	No recurrence at 30m
Yada et al. (69)	Descriptive study	Stoma closure (Japan)	2	11m, 16m	0.3 mg/kg IV	Evaluation of intestinal blood flow and postoperative bowel function (detection of ICG in stools)
Yamamichi et al. (70)	Descriptive study	Hepatoblastoma (Japan)	3	1–6 y	0.5 mg/kg IV, 3–4 days preop	Cannot detect lesions distant from liver surface and < 3 mm
Yanagi et al. (71)	Analytic cohort study (retrospective)	Biliary atresia (Japan)	10	Mean : 74.8d (48–122d)	0.5 mg/kg IV, 23h pre-op	Useful for observing biliary flow
Yokoyama et al. (72)	Descriptive study	Refractory chylous ascites (Japan)	1	2.5m	0.1 mL SC, bilateral (dorsum of each foot)	Confirmation of lymphatic duct and made treatment possible
**Pediatric neurosurgery (** ***n*** **= 12)**						
Ambekar et al. (9)	Descriptive study (retrospective)	Moyamoya disease (India)	6/13	Median: 11y	0.3 mg/kg IV	Confirmation of patency of superficial temporary artery - middle cerebral artery anastomoses
Asayama et al. (10)	Descriptive study	Skull bone tumor (Japan)	2/4	7y, 11y	0.2 mg/kg IV	Useful for tumors extending under bone surface, no recurrence at 1.5–2y
Hori et al. (36)	Descriptive study (prospective)	Moyamoya disease (Japan)	9/13	4–69y Mean age for ped patients: 12.8y +/− 5y	Not specified	Confirm patency and evaluate the anterior branch of the middle meningeal artery for preservation, good correlation with postop imaging. No recurrence at mean FU: 16m
Horie et al. (35)	Analytic cohort study (prospective)	Moyamoya disease (Japan)	14/22	Mean age for ped patients: 9.9 +/− 4y	12.5 mg IV	May have potential to predict postop hyperperfusion in Moyamoya disease
Kim et al. (43)	Descriptive study	Complex vascular neoplastic lesions (United States)	1/4	16y	25 mg IV	Confirmation of occlusion of an artery branch
Nossek et al. (49)	Descriptive study	Cerebral mycotic aneurysm (United States)	1	17y	Not specified	Intraop demonstration of complete occlusion, no recurrence at 2m
Sanchez-Fernandez et al. (54)	Descriptive study	Refractory subdural empyema (Spain)	1	11y	2.5 mg/kg IV	Preservation of viable parenchyma
Sugimoto et al. (60)	Descriptive study	Intracranial pial arteriovenous fistula (Japan)	1	3y	1.5 mg IV	Identification of fistulous shunting points, no recurrence at 6m
Takagi et al. (63)	Descriptive study	Cerebral arteriovenous malformation (Japan)	1	2y	25 mg IV	Detection and removal of residual nidus
Tanabe et al. (62)	Descriptive study (prospective)	Moyamoya disease (Japan)	8/19	Mean age for ped patients: 9.6 ± 3.1 y	5 mg IV	37% success for visualization of anterior branch of middle meningeal artery
Tsuzuki et al. (66)	Descriptive study	Endoscopic biopsy of intraventricular tumors (Japan)	3	13–14y	12.5 mg IV	Identification of tumor margins. *Limitations*: Unable to visualize the dissemination areas.
Ueba et al. (67)	Descriptive study	Spinal cord hemangioblastoma resection (Japan)	1	19m	5 mg IV	No recurrence at 1m
**Pediatric cardiac surgery (** ***n*** **= 5)**						
Kato et al. (40)	Descriptive study	Chylothorax post-coarctectomy (Japan)	1	2y	SC injection in bilateral dorsalis pedis (dose unspecified) 1	No recurrence at 6m
Kogon et al. (45)	Descriptive study (prospective)	Coronary artery re-implantations, coarctation repairs, palliative shunts, pulmonary artery reconstructions (United States)	40		1.25 mg IV (<1y), 2.5 mg IV (<16y), 5 mg IV (adults)	18/30 adequate images (60%), highest image adequacy for Blalock-Taussig shunts
Pourmoghadam et al. (52)	Descriptive study	Redo congenital cardiac surgeries (United States)	3/4		5 mg IV	Identification of aberrant coronary vascular anatomy, useful when preoperative imaging not available
Tan et al. (61)	Descriptive study	Post-Norwood procedure chylothorax (United States)	1	5w	25 mcg intradermal in dorsum L foot, 12.5 mcg dorsum R foot, 12.5 mcg dorsum L hand	*Limitations*: failure to visualize leak, postoperative patient's death (not due to ICG).
Vogt et al. (68)	Descriptive study	Arterial switch operation (Germany)	1	5d	0.05 ml/kg IV	Visualization of anatomy and flow dynamics in coronary artery system
**Pediatric plastic surgery (** ***n*** **= 12)**						
Cheng et al. (17)	Experimental study (prospective)	Primary lymphedema (Taiwan)	9	Mean: 9.2y	0.5%, 0.5 ml, SC 1st and 4th web spaces of dorsal aspect of bilateral limbs	Improves quality of life and reduces episodes of cellulitis
Drobot et al. (21)	Descriptive study	Axillary lymphatic malformation (Israel)	1	14y	0.75 mg SC, interdigits of ipsilateral hand	Successful intraoperative ICG lymphography to preserve normal lymphatic vessels
Greives et al. (30)	Descriptive study	Congenital arm and hand lymphedema (United States)	1	21m	12.5 μg intradermal, dorsum of each foot and hand	Guide treatments and evaluate lymphatic anatomy and contractile function
Hinchcliff et al. (33)	Descriptive study	Perfusion assessment of scalp closure (United States)	1	12m	2.5 mg IV	Useful to assess vascularization of flaps
Ishikawa et al. (38)	Experimental study (prospective)	Percutaneous sclerotherapy of soft-tissue venous malformations (Japan)	13/15	3–64y	0.01 mg/ml, direct injection in venous malformations	Observational depth <1 cm, additional monitor for percutaneous sclerotherapy of venous malformations *Limitations*: no fluorescence in 2 patients; no complication with ICG, but adjacent tissue ulceration (*n* = 1)
Kaneshi et al. (39)	Descriptive study	Lymphatic dysplasia (Japan)	1	248d	Not specified	Early diagnosis and severity assessment of lymphatic dysplasia
Kato et al. (41)	Descriptive study	Peri-orbital lymphangioma (Japan)	1	11m	0.005 mg SC at multiple loci	Detection of exact location of lymph vessels with minimum skin incision
Kato et al. (42)	Analytic experimental study (prospective)	Lymphatic malformations (Japan)	20	11m−10y	0.0125 mg in multiple spots, distal to lymphatic malformation	*Limitations*: depth 1 cm
Martins et al. (46)	Analytic cohort study (retrospective)	Autologous ear reconstruction (United States)	21	8.3y	5 mg IV	Decreased number of surgical revisions in cases with ICG (*p* = 0.03)
Ogata et al. (50)	Descriptive study	Lymphedema (Japan)	1/5	12y	0.2 ml Diagnogreen 0.5%SC	Guides intraoperative skin incisions et lymphaticovenular anastomoses *Limitations*: depth 2 cm, limited area (10 x 10 cm)
Shirota et al. (57)	Descriptive study	Lymphatic malformations of abdominal wall (Japan)	1	15y	0.125 mg SC and intradermal in core and 2 marginal regions of tumor	Confirmation of the extent of the tumor, complete resection of tumor, no residual fluorescence. No recurrence. *Limitations*: border not clearly visualized (ICG spillage)
Tomioka et al. (65)	Descriptive study	Congenital syndactyly (Japan)	1	1y	Not specified	Used for flap perfusion and after microanastomosis
**Pediatric urology (** ***n*** **= 3)**						
Esposito et al. (24)	Descriptive study (retrospective)	Laparoscopic Palomo varicocelectomy (Italy)	25	2–16 y	0.01 mg, left testicle	Clear detection of lymphatics in 100% patients after 20–30 s, maximum of 18 m of follow-up, no recurrence and no hydrocele
Esposito et al. (25)	Analytic experimental study (ambispective)	Laparoscopic or robotic urological procedures (varicocelectomy, nephrectomy, renal cyst deroofing) (Italy)	57	1–18y	0.3 mg/ml/kg, intra-testicular vs. IV depending on indication	Definition of surgical anatomy and vascularisation; no clear advantage in nephrectomy
Herz et al. (32)	Descriptive study	Pediatric robot-assisted laparoscopic heminephrectomy (United States)	6	0.8–13 y	1.25–2.5 mg IV	No extension of operative time
**Pediatric orthopedics (** ***n*** **= 3)**						
Aung et al. (11)	Descriptive study	Rotationplasty for sarcoma patients (Germany)	3	20–132 m	0.1 mg/kg IV	Intraoperative monitoring of limb and sciatic nerve perfusion, fluorescence seen after 20 s
Cleveland et al. (19)	Descriptive study	Trauma, circumferential open wound to posterior heel with exposed calcaneus (United States)	1/4	15 y	Not specified	Perfusion assessment during debridement
Connolly et al. (20)	Descriptive study	Trauma, Salter-Harris 2 ankle fracture with neurovascular compromise after surgery (United States)	1	13 y	4 ml IV	Guides surgical therapy for excision of devitalized tissue, aids in decision-making for major considerations such as revascularization or amputation

*d, days; kg, kilograms; ICG, indocyanine green; IV, intravenous; m, months; mg, milligrams; ml, milliliters; SC, subcutaneous; lap, laparoscopic; FU, follow-up; y, years*.

* *Only the last 15 cases were done under ICG-FA*.

† *Only the last three patients were done under ICG-FA*.

‡ *One patient with diffuse strong fluorescence underwent liver transplant 6 months after Kasai*.

### Study Quality

The risk of bias assessment can be found in Table 2. Overall, the quality of evidence supporting the use of ICG-FA in surgical pediatric patients was predominantly of low or unknown risk. An important number of included studies (*n* = 37; 58%) were at high risk of selection bias as none of them were randomized and allocation of intervention was based on the surgeon's choice. Data collection was mentioned as prospective in only 13/64 studies (14, 22, 31, 34, 44). While outcomes were generally appropriate for the study aim, 80% of included studies were at high or unclear risk of information biases either because outcomes were not objectively assessed or blinding of surgeons to the intervention was not possible. Follow-ups were inconsistently reported making it difficult to assess whether the intervention was beneficial or not in the long-term. Statistical analyses were not conducted in 40/64 studies and power calculations were lacking in all studies.

### Narrative Synthesis of the Results

#### Techniques

Most ICG-FA indications used a peripheral vein injection (*n* = 29; 45%). However, chylothorax and lymphatic/venous procedures (15, 30, 38, 40, 42, 47, 50, 51, 56, 57, 61) required subcutaneous ICG injections, and varicocelectomies (22, 24) needed direct injections into the ipsilateral testicle. Indocyanine green dose and injection timing varied according to surgical teams and indications and were adequately reported in only 29/64 (45%) studies. Other studies either overlooked stating the patient's weight, administration route, or ICG dilution making it difficult to generalize results. Injections were done intraoperatively except for specific indications including surgical procedures for primary (70) and metastatic (16, 44, 59, 64) hepatoblastoma and biliary atresia (34) as well as cholecystectomies (22, 23, 28, 31) for which ICG was injected from 18 to 72 h before the surgery.

#### Application of ICG by Pediatric Surgical Specialty

In pediatric surgery and pediatric urology, ICG-FA indications included cholecystectomy (*n* = 8) (13, 14, 22, 23, 26, 28, 31, 55), primary and metastatic hepatoblastoma (*n* = 6) (16, 44, 48, 59, 64, 70), varicocelectomy (*n* = 5) (22, 24–26, 31), nephrectomy (*n* = 5) (22, 25, 26, 28, 32), chylous leaks and ascites (*n* = 4) (15, 47, 51, 56), colorectal procedures including cloacal, anorectal malformation, and Hirschsprung reconstructions, intestinal resection for volvulus, as well as stoma closure (*n* = 4) (37, 53, 55, 69), hepatocellular carcinoma (*n* = 1) (18), abdominal mass excision (*n* = 1) (22), and tracheoesophageal fistula (*n* = 1) (58). Pediatric neurosurgeons used ICG-FA in patients with Moyamoya disease to evaluate the anatomy and confirm patency of their surgical anastomoses (*n* = 4) (9, 35, 36, 62). Tumor detection (*n* = 3) (10, 43, 66), arteriovenous malformations (*n* = 2) (63, 67), cerebral mycotic aneurysm (*n* = 1) (73), and intracranial pial arteriovenous fistula (*n* = 1) (60) were other neurosurgical indications. In pediatric cardiac surgery, ICG-FA was used for congenital cardiac surgeries (*n* = 3) (45, 52, 68) and post-operative chylothorax (*n* = 2) (40, 61). Reported indications in pediatric plastic surgery were lymphatic and venous malformations (*n* = 8) (17, 21, 30, 38, 39, 42, 50, 57) and tissue perfusion (*n* = 3) (33, 46, 65). Orthopedics indications were rare and included trauma surgeries (*n* = 2) (19, 20) and rotationplasty for patients with femoral sarcoma (*n* = 1) (11).

#### Benefits of ICG-FA

Indocyanine green appears mostly beneficial in the pediatric population for delineating vascular, lymphatic and hepatobiliary structures. A total of 57 pediatric studies (89%) reported potential benefits and successful outcomes with the use of ICG-FA. Shafy et al. (55) published a retrospective review of their general use of ICG-FA over a 2-year period, and confirmed safety of repeated injections in children (*n* = 8; 8%). After ICG was introduced, Esposito et al. (23) observed a decrease in operative time by a mean of 17 min in a 25-year retrospective study on laparoscopic cholecystectomies. ICG-FA can also confirm vascular anastomosis patency in children (22, 23, 74). In pediatric colorectal surgery, Rentea et al. (53) reported a change in the operative plan with ICG use in 4/12 (33%) patients undergoing surgery for anorectal malformations, cloaca, and Hirschsprung disease. In these four cases, the vascular supply of tissues seemed well-perfused on macroscopic inspection, but the use of ICG-FA demonstrated a poorly perfused proximal bowel which led to additional surgical steps including further resection (*n* = 2), splenic flexure mobilization (*n* = 1), and colostomy (*n* = 1). Overall, an early dehiscence of the anoplasty occurred in one patient (8%) who received intraoperative vasopressors; the remaining patients had an uneventful postoperative course. For patients who are critically ill and require lymphatic imaging, ICG-FA may represent a potential alternative because it can be performed at the patient's bedside (61). Furthermore, ICG-FA seems promising in pediatric surgical oncology. It has been reported useful for detecting pediatric tumors such as primary hepatoblastoma with or without lung and peritoneal metastases, even more when the surgery is performed by thoracoscopy and tactile sensation is not possible (16, 44, 59, 70). During Kasai procedures for biliary atresia, indocyanine green was helpful to detect bile leaks which may reduce postoperative morbidity and potentially have an impact on the postoperative normalization of hyperbilirubinemia of the patient (34). Two studies have also observed the fluorescence in patient's postoperative stools, and although this remains at an experimental stage, they found it useful to evaluate bile excretion (34) and bowel function (69). Assessment of skin flap vascularity in pediatric autologous ear reconstruction with ICG showed a decrease in surgical revisions (46).

#### Limitations of ICG-FA

Equipment access and associated costs are the main limitations of indocyanine green fluorescence angiography. While most studies found ICG useful during cholecystectomies, Bryant et al. (13) has described a case of gallbladder duplication during which intraoperative use of ICG did not provide adequate information for dissection. Although the author does not specify the timing of injection, this possibly reflects the importance of ICG administration 3 to 7 h before surgery to achieve adequate bile duct-to-liver fluorescence detection (75). Detection of fluorescence may also be limited by depth and size. Some studies reported that the detection of hepatoblastoma metastases was limited by size (<1.2 mm) (59) and distance from the surface (>3 mm) (70) of the parenchyma. One Japanese study including 250 fluorescence-positive resected lung lesions for metastatic hepatoblastoma revealed 29 false positive pulmonary lesions which were active alveolar cells and thromboses on pathological analysis (44). Another study has successfully detected hepatoblastoma pulmonary metastases of 1.2 mm at a dept of 6 mm from the lung surface (59). For lymphatic imaging, authors have also reported a limit of <1–2 cm in the depth of lymphatic vessels visualization (38, 42, 56). While most fluorescence lymphatic imaging were successful (15), ICG-FA failed to demonstrate the chylous leak in a patient who underwent a lymphatic imaging for a postoperative chylothorax. The authors hypothesized that the ICG transport toward the thorax was prevented by the high central venous pressure or lymphatics obstruction (61).

#### Adverse Events Associated With ICG

The reported incidence of adverse events is 1 out of 42 000 patients and most common reactions include urticaria, hypotension, syncope, and vasovagal reaction (2). In our review, no adverse events related to indocyanine green injection occurred in every full-text screened article. Tan et al. (61), on the other hand, described a 5-week-old patient with hypoplastic left heart syndrome who failed conservative management for bilateral chylothorax after a Norwood procedure and underwent a bedside lymphatic imaging using ICG-FA on postoperative day 18. Fluorescence lymphoscintigraphy allowed visualization of the proximal lymphatic drainage and helped to guide surgical treatment, but a few days later, the patient's clinical condition deteriorated as the abdominal ascites worsened and comfort care was offered (61). As chylothorax is a serious complication with high mortality rates, there were no adverse events associated with the ICG-FA and the patient's death was not considered related to ICG.

## Discussion

Our review and narrative synthesis on the perioperative use of indocyanine green fluorescence angiography in pediatric patients included a total of 64 articles with 664 patients. It contains all articles relevant to the surgical use of ICG-FA in pediatrics and may serve as a reference guide for pediatric clinicians. Based on our results, ICG-FA technology is a promising surgical tool and appears safe for usage in pediatric patients. However, small sample sizes and types of included articles highlight the lack of robust evidence supporting the use of indocyanine green fluorescence angiography in the pediatric population.

There was remarkable heterogeneity across included studies. Indications, dose, and outcomes of indocyanine green fluorescence angiography were extremely variable. This made comparability between articles limited and quantitative analysis not possible in our study. Fluorescence interpretation was subjective in all of the included articles which made it difficult to compare, analyze, and draw conclusions from their results. Current published studies were of low-grade evidence and did not have a prospective calculation of study sample size.

We found that the doses and administration method of indocyanine green varied depending on surgical indications. While patients' demographic and clinical factors were generally well-presented, only 24 (38%) studies adequately mentioned the dose of indocyanine green. Other articles were either missing the weight of the patient or inconsistently reported the given dose amongst patients. In the United States, the suggested dose for tissue perfusion assessment is imprecise, ranging from 1.25 to 5 mg for children older than 1 month of age (2). For hepatobiliary anatomy visualization, 2.5 mg of the fluorescent agent is recommended for patients from 12 to 17 years old. In Europe, ICG dose recommendations are also vague and only focus on original ICG indications (ophthalmologic angiography, cardiac and hepatic function studies) (76). This wide dose range may lead to inaccurate dose of indocyanine green in children which can be either too high or insufficient, both compromising the fluorescence imaging. Conversely, Shafy et al. (55) have suggested ICG dosing guidelines based on a retrospective review of their dosing practices over a 2-year period. Dosing examples for fluorescence-guided oncology surgeries with ICG were also provided by Goldstein et al. (77). However, no phase I clinical trial has been conducted to test safety and adequate dose in children. There is, therefore, no consensus on the dose and timing of indocyanine green injection even in similar indications.

ICG-FA technology provides detailed anatomical and perfusion real-time assessment for different types of surgeries, but its interpretation remains subjective as there is no threshold for adequate perfusion in both adults and children. The SPY-Q software (Stryker, United States) is a postprocessing imaging analysis software which allows an objective quantification of the fluorescence and assessment of perfusion kinetics. However, no threshold value for necrosis has been previously determined (78). A retrospective study of 90 consecutive patients undergoing esophagectomy with gastric conduit reconstruction used an arbitrary value of 75% to position their anastomosis and showed a reduction of 20% of anastomotic leaks (79). In a Japanese prospective clinical trial of 70 consecutive adult patients undergoing a reconstructed gastric tube during esophagectomy, the authors reported that perfusion of the gastric tube anastomosis is sufficient if fluorescence appears within 60 s of the injection (80). Time to fluorescence is quantitative measure that has been studied and may be promising for correlation with tissue ischemia in the future.

Alternatives to indocyanine green fluorescence angiography have been reported in the literature, but ICG-FA appears superior. Doppler, fluorescein angiography, laser fluorescence angiography, pulse oximetry, laser tissue blood flowmetry, near-infrared spectroscopy are methods that have been reviewed (81, 82). ICG-FA tissue penetration is up to 10 mm (83) which allows better visualization of deeper vessels and creates an advantage over other analogous substances such as fluorescein (1). Its selective binding to plasma proteins reduces the leak from the circulation making this molecule ideal for angiography (81). Another advantage is its quick hepatic clearance rate that allows repeated injections during a procedure (1).

Whereas pediatric surgical indications for the use of ICG-FA remain scant, it is a frequently used and valid technology in adults. In 2015, a prospective multicenter clinical trial assessed the use of ICG-FA during left colectomies and anterior resection and observed a modification of the surgical plan in 11 patients (8%) (84). No anastomotic leak occurred in these patients. Multiple other studies demonstrated the potential benefits of this fluorescent agent including intraoperative decision improvement, decreased rate of postoperative complications (85), and avoiding stomas (84). In surgical oncology, ICG-FA is mainly used to improve identification of tumors and lymph nodes. In breast cancer, the fluorescence has a similar detection rate for sentinel lymph node compared to the current combination of radioactive technetium-99 m and lymphazuran blue (83). Recent studies have proven the ability of the ICG-FA to guide lymph node dissections and sentinel lymph nodes harvestings for metastatic melanoma, papillary thyroid microcarcinoma, and early ovarian cancer (1). ICG-FA can also be applied to the identification of ureters which can be very challenging in complex gynecological and colorectal cancer surgeries (86). Indocyanine green fluorescence angiography is also frequently used for adult reconstructive surgeries to facilitate intraoperative assessment of flap viability and anastomosis perfusion (87).

In the past years, indocyanine green fluorescence imaging has gained popularity in the pediatric population. After Lau and al.'s first overview of ICG applications in Pediatric Surgery in 2009, two other reviews have been published (74, 77, 88). Goldstein et al. (77) focused on the evolving applications in surgical oncology which still seem experimental, but promising for delineating tumor margins, localizing metastases, protecting important structures around the tumor, and assisting reconstruction. Paraboschi et al. (88) have published a systematic review including 21 studies on fluorescein sodium and indocyanine green imaging in pediatric surgery. Compared to our study, they included fluorescence imaging studies at large, excluded neurosurgical, vascular, ENT, and orthopedic articles, and their last search was in January 2020. These reviews confirm the growing interest in ICG-FA in pediatric surgical specialties but compared to our review, none of them critically assessed the risk of bias of published studies. Our systematic review and narrative synthesis include the highest number of published articles (*n* = 64), a rigorous methodology and risk of bias assessment.

The ongoing study of indocyanine green fluorescence angiography remains necessary for the full development of this technology amongst the pediatric population. Multiple preliminary experiences have shown the safety and usefulness of ICG-FA in pediatric abdominal surgeries including minimally invasive surgeries (22, 26), laparoscopic Palomo varicocelectomies (24), laparoscopic cholecystectomies (89), and laparoscopic partial nephrectomies (90). Currently, efforts are being made to standardize the technique and prove the benefits of the ICG-FA technology (89, 90). A study published after the updated search of this review suggested a dose of 0.35 mg/kg of indocyanine green, 16–18 h before performing a laparoscopic cholecystectomy (89). The authors reported a 17% technical failure of intra-operative ICG fluorescence visualization (*n* = 2). Absence of fluorescence was noted in one patient with Crigler-Najjar syndrome type 2 who was taking phenobarbital. Conversely, another patient experienced liver background hyperfluorescence due to the short delay of 8 h between the ICG injection and surgery. Another pediatric study compared indocyanine green fluorescence angiography to the standard technique during laparoscopic partial nephrectomy and showed a 53 min operative time reduction with the technology (*p* = 0.001) (90).

Results of this systematic review were mainly limited by the quality of available evidence. While 13/64 studies were prospective, they did not have a randomized allocation which may lead to bias. Most included articles were either case reports or case series (*n* = 36; 56%). Known limitations of the nature of these articles are the limited ability to generalize, retrospective design, and publication bias. Unpublished articles and abstracts were excluded. We recognize that including articles with mean or median age <18 years old is a limitation in our study. However, only 16% (10/64) of included studies combined an adult and pediatric population. Another limitation is that meta-analysis was not performed due to the important heterogeneity of the articles.

## Conclusion

This systematic review and narrative synthesis bring together all relevant articles on the use of indocyanine green fluorescence angiography in pediatric patients. Indocyanine green fluorescence angiography is a safe surgical technology when used in the pediatric population. Pediatric applications of ICG currently remain limited, but there is an overall important increase in clinical applications of ICG-FA. Larger, controlled clinical studies are required to determine and standardize the adequate dose of ICG and timing of administration for various indications in children. Multicentric collaboration will likely be needed to accomplish this. The Delphi technique could be applied to gain consensus on indications and doses of ICG -FA in pediatric surgical specialties and improve data comparison.

## Data Availability Statement

The original contributions presented in the study are included in the article/supplementary material, further inquiries can be directed to the corresponding author/s.

## Author Contributions

AL-N, PD, MK, CF, and NP contributed to conception and design of the study. AL-N organized the database. AL-N and MO'N analyzed all included studies. AL-N wrote the first draft of the manuscript. All authors contributed to manuscript revision, read, and approved the submitted version.

## Conflict of Interest

The authors declare that the research was conducted in the absence of any commercial or financial relationships that could be construed as a potential conflict of interest.

## Publisher's Note

All claims expressed in this article are solely those of the authors and do not necessarily represent those of their affiliated organizations, or those of the publisher, the editors and the reviewers. Any product that may be evaluated in this article, or claim that may be made by its manufacturer, is not guaranteed or endorsed by the publisher.

## Abbreviations

ICG: indocyanine green
ICG-FA: indocyanine green fluorescence angiography
MINORS: methodological index for non-randomized studies
PRISMA: preferred reporting items for systematic reviews and meta-analyses.
